# Molecular Properties of Water-Unextractable Proteoglycans from *Hypsizygus marmoreus* and Their *in Vitro* Immunomodulatory Activities

**DOI:** 10.3390/molecules17010207

**Published:** 2011-12-27

**Authors:** Hong Hui Bao, Mehdi Tarbasa, Hee Mun Chae, Sang Guan You

**Affiliations:** 1 Department of Marine Food Science and Technology, Gangneung-Wonju National University, 120 Gangneung Daehangno, Gangneung, Gangwon, 210-702, Korea Email: honghui_bao@163.com (H.H.B.); mtabarsa@yahoo.com (M.T.); 2 Climate Change Research Institute of Korea, Chuncheon-si, Gangwon-do, 200-939, Korea

**Keywords:** *Hypsizygus marmoreus*, proteoglycan, immunomodulation activity, molecular conformation, glycosidic linkages

## Abstract

Four proteoglycans were sequentially extracted from *Hypsizygus marmoreus* using 0.1 M NaOH (alkali-soluble proteoglycans [F1] and alkali-insoluble proteoglycans [F3]) and 0.1 M HCl (acid-soluble proteoglycans [F2] and acid-insoluble proteoglycans [F4]), and their structures and immunomodulatory activities were investigated. The proteoglycans were found to contain carbohydrates (19.8–82.4%) with various amounts of proteins (7.7–67.3%), and glucose was the major monosaccharide unit present, along with trace amounts of galactose. The molecular weights (Mw) and the radius of gyration (Rg) of these proteoglycans showed ranges of 16 × 10^4^–19,545 × 10^4^ g/mol and 35–148 nm, respectively, showing significant variations in their molecular conformations. The backbones of F1 and F2 were mainly connected through α-(1®3), (1®4) and b-(1®6)-glycosidic linkages with some branches. The F1 and F2 proteoglycans significantly stimulated Raw264.7 cells to release nitric oxide (NO), prostaglandin E2 (PGE_2_) and various cytokines, such as IL-1β, TNF-α and IL-6 by inducing their mRNA expressions.

## 1. Introduction

Edible mushrooms have been widely used for millennia as a food delicacy, a source of important medicinal substances and as nutritional supplements. Among the various constituents, such as essential amino acids, proteins, carbohydrates, minerals and vitamins; polysaccharides have attracted considerable attention from biochemical and nutritional researchers because of their potential importance as functional foods and medicinal ingredients. Polysaccharides from *Lentinus edodes*, *Schizophyllum commune* and *Coriolus versicolor* are well known to have strong antitumor activity and are commercially available as anticancer supplements [1,2,3]. It has been reported that the soluble polysaccharides from *Tricholoma* sp. improved the proliferation of peritoneal macrophages in mice and inhibited tumor cell growth, possibly through the enhancement of the host immune system [4]. In contrast, the polysaccharides from *Pleurotus ostreatus* and *Pleurotus tuber-regium* directly suppressed the growth of cancer cell lines (HT-29 and HL-60) by upregulating the pro-apoptotic molecules, Bax and cytosolic cytochrome-*c* [5,6].

It is well known that the antitumor and immunomodulatory activities of the polysaccharides are closely related with their primary and secondary structures [7]. According to Demleitner *et al*. [8], the β-(1→3)-glycosidic linkage was proposed to be essential for antitumor activity in the study of curdlan and licheman derivatives. However, there are various antitumor polysaccharides from mushrooms having other glycosidic linkages, such as α-(1→4)-glycoside [9], β-(1→6)-glycoside, α-(1→6)-heteroglycoside [10]. Maeda and Watanabe [11] mentioned the importance of secondary structures of the polysaccharides, reporting that the triple helical conformation led to higher stimulation of the T-cell mediated immune responses than did single chains of β-D-glucan. The molecular weight of mushroom polysaccharides has also been suggested to be an important factor to its bioactivities, reporting that high molecular weight glucans ranging from 500–2,000 kDa were more effective than those of lower molecular weight [12]. Although there is much controversy surrounding the relationship between molecular structures and biological activities, it is still important to characterize the primary and higher-order structures of polysaccharides and to correlate the structures with those bioactivities.

Hot water-extraction has been a popular approach for extracting polysaccharides from fungal cell walls, but it should be noted that considerable quantities of water-unextractable polysaccharides with various structures and bioactivities were also found [13,14]. In our previous work, we showed that water-soluble polysaccharides from *Hypsizygus marmoreus* exhibited strong immunomodulation, possibly with antitumor activity. However, the structural characteristics and biological activities of the water-unextractable polysaccharides from *H. marmoreus* are currently not known. Therefore, in this study, water-unextractable proteoglycans were sequentially extracted, from the residue after water-extraction of *H. marmoreus*, using dilute alkali and acid. The purpose of this study was to investigate the structural characteristics of the water-unextractable proteoglycans from *H. marmoreus* and to investigate their immunomodulatory activities. 

## 2. Results and Discussion

### 2.1. Chemical Composition Analysis

When the yield and chemical composition of water-unextractable proteoglycans (F1, F2, F3 and F4) were investigated, the largest amount was observed in the F1 fraction (7.5%) collected from the water-insoluble residue, while relatively small amounts of F3 and F4 fractions (0.3 and 0.4%) were obtained. The water-insoluble residue also contained a substantial amount of the F2 fraction (1.9%). The total yield (10.1%) of water-unextractable proteoglycans was a little lower than that of water-extractable proteoglycans (12%), as reported in our previous study [15]. The lower yield of the water-unextractable proteoglycans was probably due to the use of dilute alkali and acid (0.1 N) for the extraction of the proteoglycans to prevent their molecular degradation. Similar results were observed in a report by Hromadkova *et al*. [16]; in which, the total yield of water-unextractable polysaccharides from *Salvia officinalis* was significantly lower than that of the water-extractable polysaccharides.

The constituents of F1 and F2 were mainly carbohydrates (62.8% and 82.4%, respectively) containing considerable amounts of proteins (16.5% and 7.7%, respectively). The proteins were retained in F1 and F2 fractions after repeated Sevag treatments, suggesting that the proteins in F1 and F2 might be covalently bound to the polysaccharides. When compared with the carbohydrate (55.8%) and protein (23.0%) contents of the water-extractable polysaccharides reported in our previous study [15], the water-unextractable proteoglycans contained higher quantities of carbohydrates and lower quantities of proteins. However, significant amount of proteins (39.7% and 67.3%, respectively) were included in alkali and acid insoluble (F3 and F4) fractions. This suggested that the solubility of the water-unextractable proteoglycans might be related to protein content. It is likely that the proteins in F3 and F4 were initially soluble at alkaline conditions but became insoluble when the pH of solution became neutral, possibly because of their intra and/or inter-molecular interactions. This pH change seemed to cause the aggregation of F3 and F4, followed by their precipitation. Similar results were also observed in the study of proteoglycans, which were initially soluble in 0.04 M NaOH but became insoluble at neutral pH due to the intra and/or inter-molecular interactions of proteins [17].

Monosaccharide composition analysis revealed that glucose was the major sugar in the water-unextractable proteoglycans and a considerable amount of galactose (8%) was only found in the F4 fraction. As reported in our previous study [15], glucose was also a major sugar in the water-extractable polysaccharides with slightly higher quantities of galactose (11.6%), which implied that the polysaccharides of the fruiting body from *H. marmoreus* were relatively homogeneous polysaccharides mainly consisting of glucose as a major monomer unit. Other monosaccharides such as mannose, xylose and fucose were not detected in the current study.

### 2.2. Molecular Characteristics of Proteoglycans

**Figure 1:**
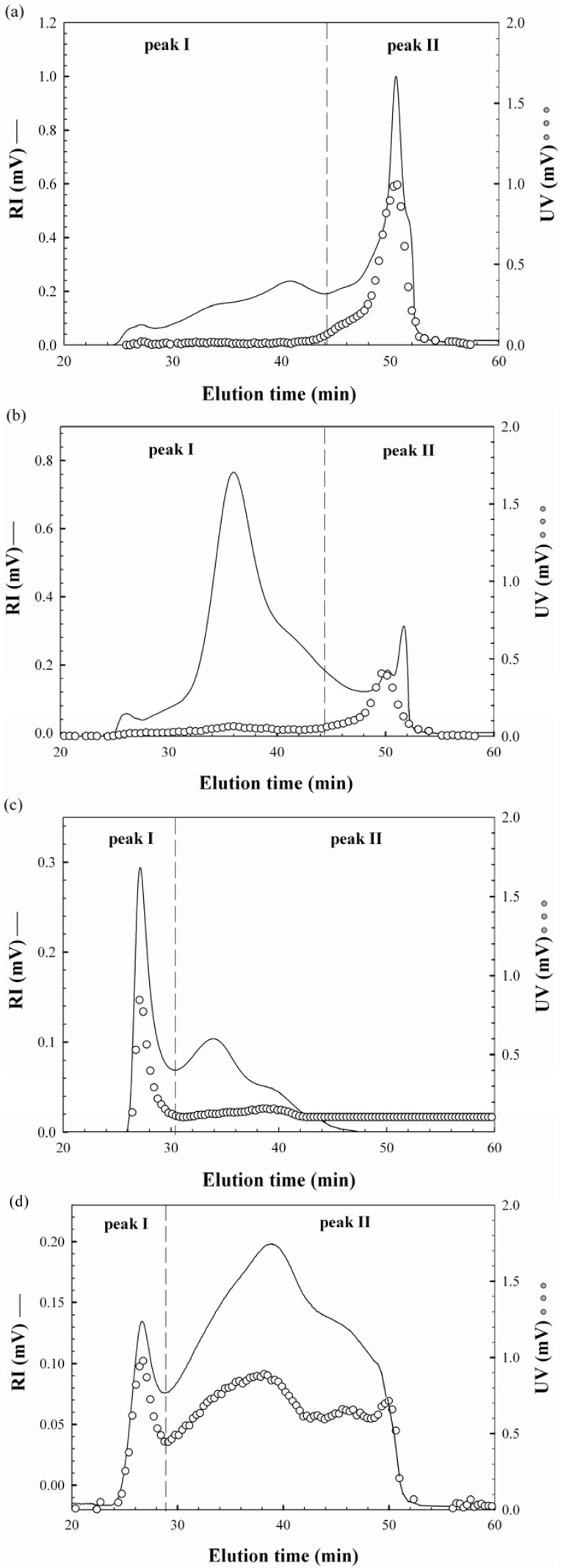
RI and UV chromatograms of the water-unextractable proteoglycans (**a**) F1, (**b**) F2, (**c**) F3 and (**d**) F4 from *H. marmoreus*.

The UV and RI superimposed chromatograms for the water-unextractable proteoglycans are shown in Figure 1. F1 and F2 were eluted from the SEC column between the elution times of 24.2 and 52.1 min having major, distinct peak areas at 50.5 and 36.2 min, respectively, with some minor peaks, which indicated that both proteoglycans had heterogeneous polymer distributions (Figure 1a,b). The ratios of the peaks were significantly different between F1 and F2. Similar distribution ratios of high and low molecular weight fractions were observed for F1 while most polysaccharides were included in higher molecular weight fractions for F2. The UV chromatogram indicated that proteins were mostly included in peak II between the elution times of 44.2 to 52.1 min. Table 1 showed that the M_w_ values of the peaks were 753 × 10^4^ g/mol and 16 × 10^4^ g/mol for F1 and 259 × 10^4^ and 25 × 10^4^ g/mol for F2, respectively. This is the first report for the M_w_ of the water-unextractable proteoglycans from *H. marmoreus*. In the study of water-unextractable polysaccharides from other mushrooms including *P. tuber-regium*, *P. ostreatus*, *P. eryngii* and *Laetiporus sulphureus*, the M_w_ values ranged from 5.8 × 10^4^ g/mol to 290 × 10^4^ g/mol, exhibiting significant variations [18,19,20]. These considerable variations in the M_w_ might be attributable to differences, not only in the mushroom species but also in the extraction, purification and analysis methods as well as the different life cycles of the mushrooms. The radius of gyrations (R_g_) for F1 and F2 were also calculated from the peaks to estimate the approximate sizes of the proteoglycans (Table 1). The R_g_ values of the two peaks of F1 and F2 were 73 and 136 nm, and 50 and 148 nm, respectively. Although peak II had significantly lower M_w_, their R_g_ values were greater than those of peak I. This was probably due to differences in the molecular conformation of the proteoglycans, suggesting that the polysaccharides of peak II might exist in an extended conformation while those of peak I in a more compact conformation, possibly through the intra-molecular interactions. The molecular conformation was also assumed by the specific volume for gyration (SV_g_) calculated by the values of M_w_ and R_g_, as reported by You and Lim [21] based on the following equation:

SV_g_ = 4/3π(R_g_ × 10^8^)^3^/(M_w_/N) = 2.522 R_g_^3^/M_w_

**Table 1:** Weight average molecular weights (M_w_), radius of gyration (R_g_) and specific volume for gyration (SV_g_) of proteoglycans (F1, F2, F3 and F4) extracted from *H. marmoreus*.

Sample	Ratio (%)		M_w_ × 10^4^ (g/mol)		R_g_ (nm)		SV_g_ (cm^3^/g)	
	Peak I	Peak II	Peak I	Peak II	Peak I	Peak II	Peak I	Peak II
F1	48.0	52.0	753 ± 8	16 ± 1	73 ± 1	136 ± 1	0.133 ± 0.005	40.5 ± 2.06
F2	82.0	18.0	259 ± 4	25 ± 1	50 ± 1	148 ± 6	0.124 ± 0.008	32.8 ± 1.66
F3	41.1	58.9	8428 ± 48	1797 ± 18	37 ± 0	35 ± 1	0.002 ± 0.000	0.006 ± 0.000
F4	10.0	90.0	19,545 ± 98	444 ± 5	123 ± 6	40 ± 2	0.024 ± 0.001	0.037 ± 0.001

nd: not detected.

**Figure 2:**
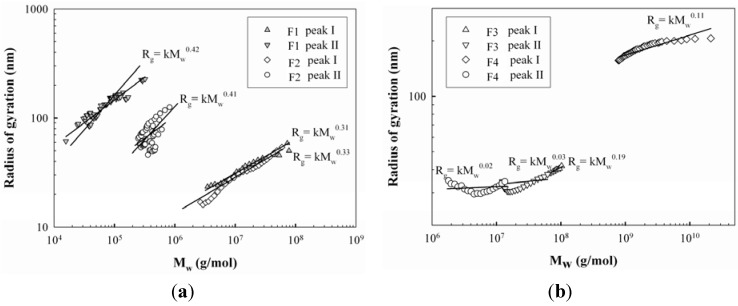
The plot of weight average molecular weight (M_w_) vs radius of gyration (R_g_) of proteoglycans: F1, F2, F3, and F4.

The units for SV_g_, M_w_ and R_g_ were cm^3^/g, g/mol and nm, respectively. In the equation, N is Avogadro’s number (6.02 × 10^23^/mol). The SV_g_ value is inversely proportional to the degree of molecular compactness, providing the theoretical gyration volume per unit of molar mass, which gives the mass-based molecular density of proteoglycans. It was shown in Table 1 that the SV_g_ values of the two peaks were 0.133 and 40.5 for F1 and 0.124 and 32.8 for F2, suggesting that the proteoglycans in peak II had less compact and more expanded conformational structures than those in peak I. This was also implied in the plot of M_w_ and R_g_ from Figure 2a that showed the polysaccharides from peak I of F1 and F2 had a relatively compact sphere conformation (α = 0.31 and α = 0.33), while those from peak II were in the conformation of a random coil (α = 0.42 and α = 0.41).

The RI and UV chromatograms of F1 and F2 are shown in Figure 1c,d. Two major peaks were observed, but their elution profiles were different from those of F1 and F2, showing distinct high molecular weight fraction peaks. Similar distribution ratios of high and low molecular weight fractions were also observed in F3, but most polysaccharides in F4 were eluted in peak II. The UV chromatograms indicated that proteins of F3 were mainly included in peak I between the elution times of 26 to 30 min, while those of F4 were contained in both peaks. This is considerably different from the F1 and F2 results, in which most proteins were eluted at peak II. The M_w_ values of the peaks were 8,428 × 10^4^ g/mol and 1,797 × 10^4^ g/mol for F3 and 19,545 × 10^4^ g/mol and 444 × 10^4^ g/mol for F4, respectively (Table 1), indicating that their M_w_ values were significantly greater than those of F1 and F2, except in peak II of F4. The R_g_ values of the two peaks of F3 and F4 were 37 and 35 nm, and 123 and 40 nm, respectively, with the ranges of SV_g_ values from 0.002 to 0.037, which indicated that the proteoglycans were in the more compact conformations than those of F1 and F2. This was also demonstrated in the plot of M_w_ and R_g_ (Figure 2b) that the polysaccharides of F3 and F4 had more compact sphere conformation (α = 0.03~0.19) than those of F1 and F2 (α = 0.31~0.41). It was not clear why the conformations of the proteoglycans between alkali/acid-soluble and alkali/acid-insoluble proteoglycans were considerably different. This is likely to be due to differences in protein content in these fractions because the ionic charges of the proteins seemed to be significant, leading to considerable intra-molecular interactions. Therefore, the amount of proteins included in the proteoglycan fractions would be an important factor contributing to proteoglycan conformation. Consequently, higher protein contents in F3 and F4 appeared to be enough to induce greater intra-molecular interactions of the polymers, resulting in more compact conformations. Furthermore, the presence of proteins at the high molecular weight fractions (peak I of Figure 1c,d), either bound or non-bound, might facilitate the molecular foldings of the high M_w_ proteoglycans, which might also make a contribution on the molecular compactness of F3 and F4. Moreover, Bandtlow and Zimmermann [22] reported, that the molecular conformation of a proteoglycan, called glypican, might be influenced by the intra-molecular interactions of proteins conferring a rather compact shape on the proteoglycans.

### 2.3. Immunomodulatory Activity of Water-Unextractable Proteoglycans

**Figure 3:**
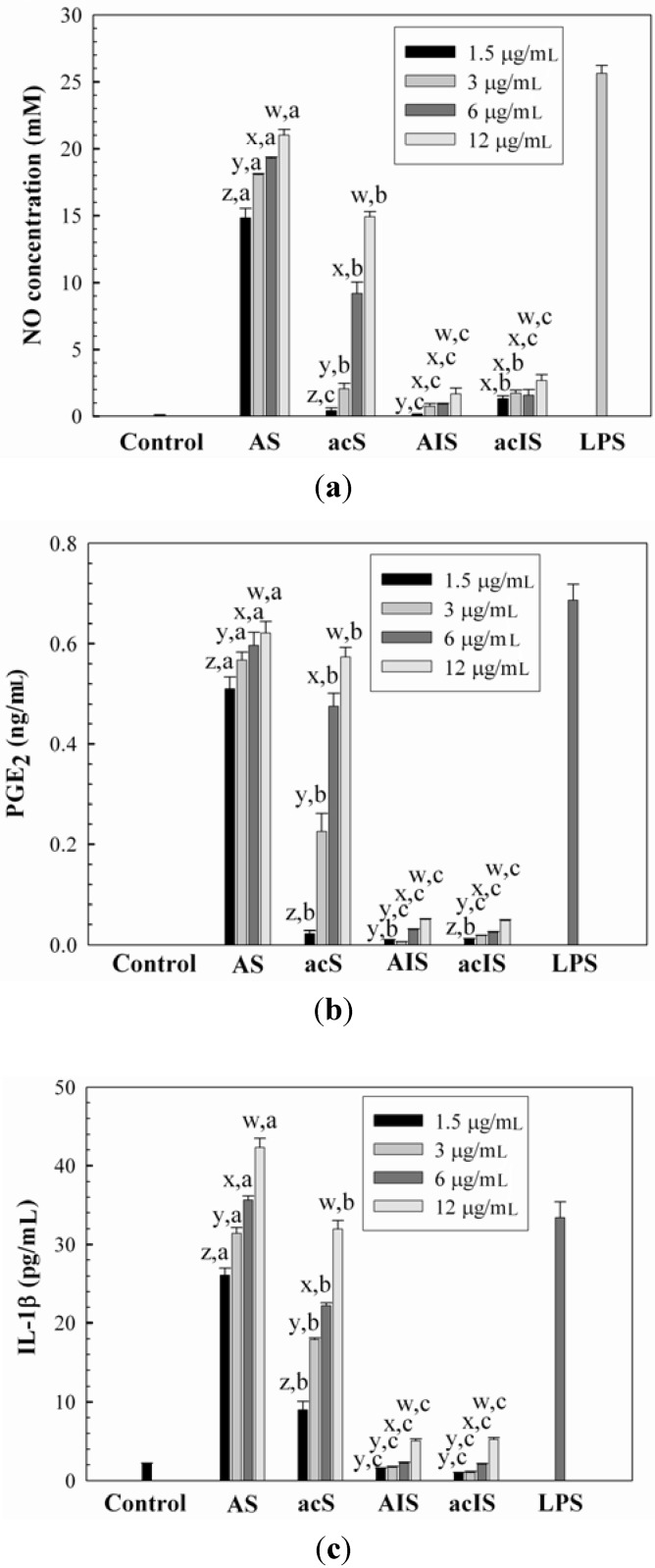
The effects of water-unextractable proteoglycans (F1, F2, F3 and F4) on (**a**) NO, (**b**) PGE_2_ and (**c**) IL-1β production. Cells were treated with the proteoglycans (1.5, 3, 6 and 12 mg/mL) or LPS (1 μg/mL) for 24 h. ^x, y, z^ indicate a significant difference (*p* < 0.01) between the concentrations of the crude and individual fractions; ^a, b, c, d^ indicate a significant difference (*p* < 0.01) between the crude and fractions at each concentration.

A murine macrophage cell line, Raw264.7 cell, has been known to release cytokines in response to the addition of lipopolysaccharide, LPS, and this system has been used to determine the immunomodulating activities of compounds [23]. In the current study, the effect of water-unextractable proteoglycans on immunostimulation was determined using Raw264.7 cells by determining the released amount of NO, IL-1β and PGE_2_. The cytotoxic effect of the proteoglycans on the proliferation of Raw264.7 cells was evaluated at concentrations of 1.5–12 μg/mL. It was shown that the proteoglycans did not considerably affect the proliferation of Raw264.7 cells, suggesting that the proteoglycans were not toxic to Raw264.7 cells over the concentration range tested in this study (data not shown). The NO production capacities of the water-unextractable proteoglycans at concentrations of 1.5–12 μg/mL, expressed as the amount of NO released from Raw264.7 cells, are shown in Figure 3a. Lipopolysaccharide (LPS, 1 μg/mL) was used as a positive control. It was shown that F1 significantly enhanced NO production (14.8–21.0 μM) in a dose-dependent manner, comparable to the amount of NO produced by the positive control, LPS. This indicated that F1 could be a strong stimulant of Raw264.7 cells. The significant dose-dependent increase of NO release (0.4–14.9 μM) was also shown in F2. On the other hand, the levels of NO production by F3 and F4 were found to be very low (<2.7 μM), indicating a weak stimulatory activity on Raw264.7 cells. The release of NO from stimulated macrophages is an important molecule in the regulation of the immune system against tumors [24]. Therefore, the substantial increase in NO released from murine macrophages in the presence of F1 and F2 proteoglycans suggested that these proteoglycans could act as biological response modifiers in host defense mechanism and thus could improve the host immune response. It is not clear why F1 and F2 proteoglycans exhibited considerably higher NO releasing capacity than F3 and F4 proteoglycans. It has been reported that the immunomodulatory activities of polysaccharides are extensively dependent on their chemical composition, molecular weight, conformation, glycosidic linkage type and degree of branching, *etc*. [25]. Among the various factors reported, in the current study, it was likely that the different molecular conformation derived from various protein contents might be related with their immunostimulating capacity. F1 and F2 with more extended and loose conformations seemed to have better binding capacity on the cell surface receptors than F3 and F4 that had compact conformations that seemed to cause greater stimulation of Raw264.7 cells. This may be explained by the observation that in proteoglycans that adopt a compact conformation via intra-molecular interactions, the charged groups (mostly from proteins) available to bind the cell receptors may be buried inside the chains, and this causes a reduction in the binding capacity to the cell surface. On the other hand, if the proteoglycans were in the loose and entangled conformation, most charged groups existing in the chain are likely to be available to bind the cell surface, thus effectively enhancing the stimulation of the cells. In the study of polysaccharides having anionic sulfate groups, the molecular conformation was also suggested as a major factor that determined which polysaccharides inhibited the growth of cancer cells through its binding capacity on the receptor of the cell surface [26].

Similar trends were observed in the releases of PGE_2_ and IL-1β from Raw264.7 cells by the water-unextractable proteoglycans (Figure 3b,c). F1 and F2 exhibited considerably higher amounts of PGE_2_ (0.57–0.62 ng/mL at 12 μg/mL) and IL-1β (32.0–42.0 pg/mL at 12 μg/mL) production in a dose-dependent manner than F3 and F4, which was comparable to the levels of PGE_2_ and IL-1β released by LPS.

**Figure 4:**
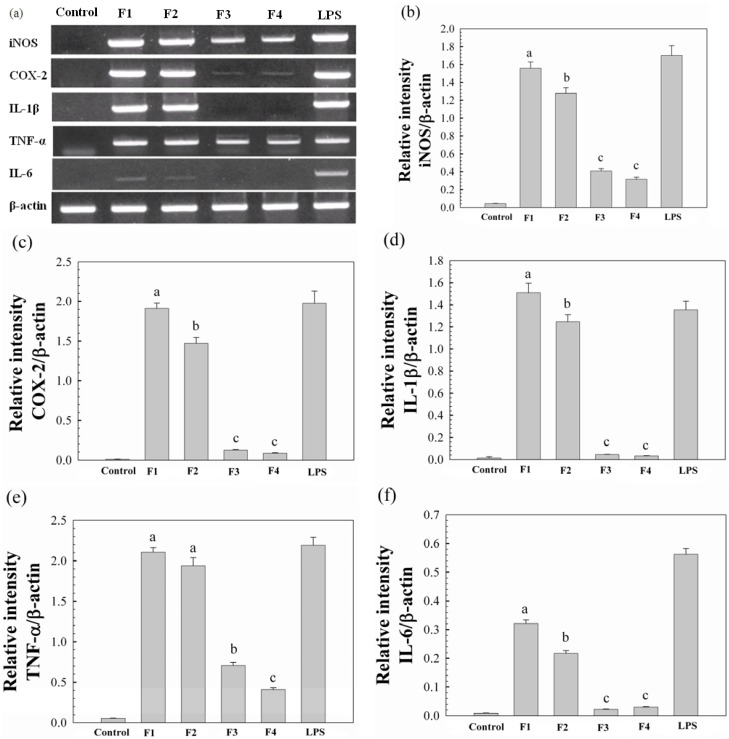
The effects of the water-unextractable proteoglycans (F1, F2, F3 and F4) on iNOS, COX-2, IL-1β, TNF-α and IL-6 expression in Raw264.7 cells. (**a**) Representative PCR gels stained with ethidium bromide. Cells were incubated with LPS (1 μg/mL) and proteoglycans (12 μg/mL) for 18 h. Total RNA was isolated, and mRNA expression was determined by RT-PCR; Graphic analysis of the PCR product for RNA levels of iNOS (**b**), COX-2 (**c**), IL-1β (**d**), TNF-α (**e**) and IL-6 (**f**). All data are presented as the mean ± standard deviation (n = 3).

We also evaluated whether the release of NO, PGE_2_ and IL-1β from Raw264.7 cells as a result of the proteoglycans, might have an impact on mRNA expression of iNOS, COX-2 and IL-1β (Figure 4a–d). The mRNA expression patterns regulated by these proteoglycans at a concentration of 12 μg/mL were determined by agarose gel analysis of RT-PCR products with primers of iNOS, COX-2 and IL-1β mRNAs, and the levels of mRNA expression were obtained by the densitometric analysis. As shown in Figure 4, Raw264.7 cells treated with F1 and F2 produced more mRNAs than F3 and F4, which was in a good agreement with the results shown in Figure 3. Moreover, it was shown in Figure 4a,e,f that F1 and F2 also significantly induced mRNA expression of TNF-α and IL-6. It has been reported that activated macrophages can suppress tumor cell growth through the release of TNF-α, which is responsible for killing cancer cells [27]. In addition, IL-6 has been regarded as a major immune mediator [28]. Therefore, the above results suggested that F1 and F2 might modulate host immune function by releasing various chemokines and/or cytokines, and thus could be potentially applied as a therapeutic agent for cancer treatment.

### 2.4. Glycosidic Linkage Analysis of F1 and F2

The polysaccharides of the most immuno-enhancing proteoglycans (F1 and F2) were permethylated according to Hakomori [29] followed by hydrolysis, reduction and acetylation of the partially methylated alditols to further investigate their structural characteristics, especially the glycosidic linkage types. Table 2 shows the types and ratios of glycosidic linkages of the polysaccharides determined by GC/MS.

**Table 2:** Glycosidic linkage analysis of the constituent sugars of the F1 and F2 fractions originated from *H. marmoreus*.

Methylated sugars ^a^	Retention time (min)	Mass fragmentation (*m/z*)	Glycosidic linkage	Peak area (%)	
				F1	F2
2,3,4,6-Me_4_-Glc	9.64	43, 59, 71, 87, 102, 118, 129, 145, 162, 174, 205	Glc-(1→	12.9	21.2
2,4,6-Me_3_-Glc	11.29	43, 59, 71, 87, 101, 118, 129, 161, 234	→3)-Glc-(1→	8.7	29.0
2,3,6-Me_3_-Glc	11.47	43, 59, 71, 87, 102, 118, 129, 142, 162, 173, 233	→4)-Glc-(1→	57.0	19.1
2,3,4-Me_3_-Glc	11.77	43, 59, 71, 87, 102, 118, 129, 143, 162, 173, 189, 233	→6)-Glc-(1→	8.4	10.2
2,3-Me_2_-Glc	13.44	43, 59, 74, 85, 102, 118, 127, 142, 162, 201, 261	→4,6)-Glc-(1→	6.2	2.9
2,4-Me_2_-Glc	13.62	43, 59, 74, 87, 101, 118, 129, 139, 160, 174, 189, 234	→3,6)-Glc-(1→	6.8	17.6
2,3,4-Me_3_-Gal	12.26	43, 59, 71, 87, 102, 118, 129, 142, 162, 173, 189, 233	→6)-Gal-(1→	nd	nd

^a^ 2,3,4,6-Me_4_-Glc represented 1,5-di-*O*-acetyl-2,3,4,6-tetra-*O*-methyl glucitol, *etc*.

**Figure 5:**
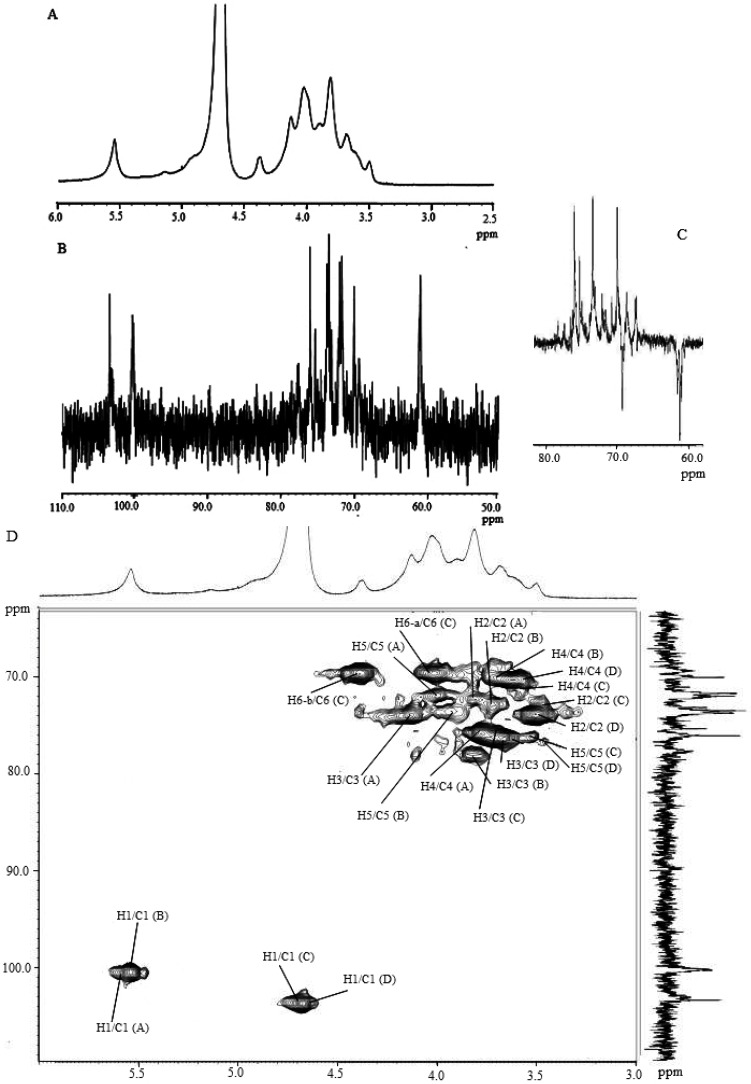
(**A**) ^1^H-NMR spectrum (600 MHz, D_2_O, 50 °C); (**B**) ^13^C-NMR spectrum (150 MHz, D_2_O, 50 °C); (**C**) DEPT-135-^13^C (150 MHz, D_2_O, 50 °C); and (**D**) HMQC spectrum (600 MHz, D_2_O, 50 °C) of F1 in aqueous solution.

It was shown that 2,3,6-Me_3_-Glcp was the most abundant component of the permethylated and acetylated derivatives from F1, which suggested that the backbones of the F1 polysaccharides might be mainly glucose molecules connected by 1,4-glycosidic linkages. In addition, F1 included small amounts of 2,4,6-Me_3_-Glcp and 2,3,4-Me_3_-Glcp, implying that the backbone was also connected through 1,3 or 1,6-glycosidic linkages. Furthermore, the presence of 1,4,6 or 1,3,6-linked glucose residues (2,3-Me_2_-Glc and 2,4-Me_2_-Glc) indicated that some branches may exist along the backbone. The total percentage of terminal glucose was 12.9%, which matched well with the branched portions (4,6- and 3,6-linked glucose: 13.0%). The ratios of the glycosidic linkages of 1→, 1→4, 1→3, 1→6, 1→4,6, 1→3,6 were calculated to 0.23:0.15:1.00:0.15:0.11:0.12. The presence of mixed glycosidic linkages such as 1→4, 1→3 and 1→6 in the F1 backbone assumed that it had stair-like structural features as depicted in Figure 5. The glycosidic linkages of F1 were also inferred from ^1^H- and ^13^C-NMR spectra (Table 3, Figure 5). Based on the available data in the literature [30], the resonance from the ^1^H-NMR spectrum at 5.52 ppm was attributed to a proton from anomeric carbons in the α configuration. The appearance of the anomeric carbon signals at 100.2 and 100.0 ppm also indicated that the (1→4)-D-Glcp and (1→3)-D-Glcp were both in the α-anomeric configuration, suggesting the residue appeared to be an α-glucan (Table 3). The carbon signal at 75.3 ppm should be C-4 of the (1→4)-D-Glcp, which was shifted about 6 ppm downfield compared with the resonance of standard glycoside due to the effect of the glycosylation [31]. On the other hand, the C-3 signal at 77.7 ppm of the (1→3)-D-Glcp appeared to be at 5 ppm downfield compared with that of C-3 of standard glycoside. The carbon signals at 73.7, 71.9, 71.7 and 61.0 ppm corresponded to C-3, C-2, C-5 and C-6 of the (1→4)-α-D-Glcp, respectively. The other signals at 73.1, 72.1, 70.0 and 61.3 ppm corresponded to C-5, C-2, C-4 and C-6 of the (1→3)-α-D-Glcp, respectively. Furthermore, another anomeric proton at 4.68 ppm was observed with a chemical shift of C-1 at 103.3 ppm, indicating the b-configuration of glucose residue. The carbon signal at 69.3 ppm should be C-6 of the (1→6)-D-Glcp, which was shifted about 8 ppm downfield compared with the resonance of the standard glycoside. This was confirmed from the inverted signal of the DEPT spectrum [Figure 5(c)]. The signals at 76.3, 76.1, 74.0 and 70.0 ppm corresponded to C-5, C-3, C-2, C-4 of the (1→6)-b-D-Glcp. In addition, the carbon signals of 76.6, 76.0, 73.8, 70.0 and 60.9 ppm were assigned to C-5, C-3, C-2, C-4 and C-6 of (1→)-b-D-Glcp, which suggested as a terminal glucose residue. These were in good agreement with the GC/MS data. However, the other glycosidic linkages suggested in the GC/MS data, such as 1→3,6 and 1→4,6 (branching points) were not able to be determined at the NMR spectrum due to complex and overlaid peaks.

**Table 3:** ^1^H- and ^13^C-NMR spectral data of F1 and F2 in D_2_O. ^1^H and ^13^C chemical shifts (relative to external TSP, =0 ppm) for F1 and F2 in aqueous solution.

Sample	Residue	C-1/H-1	C-2/H-2	C-3/H-3	C-4/H-4	C-5/H-5	C-6/H-6a, H-6b
F1	A α (1→4) Glu	100.2/5.52	71.9/3.81	73.7/4.13	75.3/3.77	71.7/3.99	61.0/3.89, 4.05
	B α (1→3) Glu	100.0/5.51	72.1/3.72	77.7/3.81	70.0/3.60	73.1/3.90	61.3/3.88, 4.00
	C β (1→6) Glu	103.1/4.68	74.0/3.47	76.1/3.71	70.0/3.58	76.3/3.52	69.3/4.01, 4.37
	D β (1→) Glu	103.3/4.68	73.8/3.49	76.0/3.67	70.0/3.58	76.6/3.45	60.9/3.51, 3.85
F2	A α (1→4) Glu	100.2/5.53	71.9/3.80	73.7/4.12	76.1/3.75	71.6/3.99	61.0/3.89, 4.05
	B α (1→3) Glu	100.0/5.52	72.4/3.73	77.5/3.80	70.2/3.60	73.5/3.90	61.3/3.88, 4.00
	C β (1→6) Glu	103.1/4.68	74.0/3.55	76.1/3.69	70.0/3.58	76.3/3.52	69.3/4.01, 4.37
	D β (1→) Glu	103.3/4.67	73.9/3.49	76.3/3.67	70.0/3.58	76.5/3.5	60.9/3.51, 3.85

It was shown in Table 2 that the major component of the derivatives from F2 was 2,4,6-Me_3_-Glc*p*, indicating that the backbone of F2 might be mainly connected with 1,3-glycosidic linkages. In addition, F2 included considerable amounts of 2,3,6-Me_3_-Glc*p* and 2,3,4-Me_3_-Glc*p*, indicating the presence of 1,4 and 1,6-glycosidic linkages in the backbone. The mixed glycosidic linkages (1→4, 1→3 and 1→6) in F2 implied that it also had stair-like structural features. The ratios of the glycosidic linkages of 1→, 1→4, 1→3, 1→6, 1→4,6, 1→3,6 were calculated to 0.73:0.65:1.00:0.35:0.10:0.60. This result indicated that the glycosidic linkages of the F2 backbone were more heterogeneous than those of the F2 backbone, suggesting that the backbone structures of the two were significantly different. The resonances from the ^1^H-NMR spectrum at 5.52 and 5.53 ppm were attributed to protons from anomeric carbons at the α configuration. The appearance of the anomeric carbon signals at 100.2 and 100.0 ppm indicated that the (1→4)-D-Glc*p* and (1→3)-D-Glc*p* were both at the α-anomeric configuration (Table 3). The carbon signals at 76.1, 73.7, 71.9, 71.6 and 61.0 ppm corresponded to C-4, C-3, C-2, C-5 and C-6 of the (1→4)-α-D-Glc*p*, respectively. The signals at 77.5, 73.5, 72.4, 70.2 and 61.3 ppm corresponded to C-3, C-5, C-2, C-4 and C-6 of the (1→3)-α-D-Glc*p*, respectively. Another anomeric proton at 4.68 ppm was observed with the chemical shift of C-1 at 103.1 ppm, indicating the b-configuration of glucose residue. In addition, the carbon signal at 69.3 ppm should be C-6 of the (1→6)-D-Glc*p* by the downfield shift of 8 ppm, which was also confirmed by the DEPT spectrum. The signals at 76.3, 76.1, 74.0 and 70.0 ppm corresponded to C-5, C-3, C-2, C-4 of the (1→6)-b-D-Glc*p*. The carbon signals of 76.5, 76.3, 73.9, 70.0 and 60.9 ppm were assigned to C-5, C-3, C-2, C-4 and C-6 of (1→)-b-D-Glc*p*, suggesting as a terminal glucose residue. It was also difficult to determine the other glycosidic linkages (1→3,6 and 1→4,6) suggested in GC/MS data at the NMR spectrum due to the complexity of the peaks. According to Maeda and Chihara [32], the b-(1→3) and (1→6) glycosidic linkages of the polysaccharides from *Lentinula edodes* was a major factor for its strong anticancer activity. However, in the current study, the most active proteoglycans, F1 and F2, had considerably different glycosidic linkages, suggesting that the type of glycosidic linkages might not be a major factor affecting their biological activity.

## 3. Experimental

### 3.1. Materials

The dried and milled fruiting body of *Hypsizygus marmoreus* was kindly provided by HaeSong Bio Co. (Gangneung, Korea) and stored at −20 °C prior to the extraction of proteoglycans.

### 3.2. Preparation of the Alkali and Acid Extractable Proteoglycans

**Figure 6:**
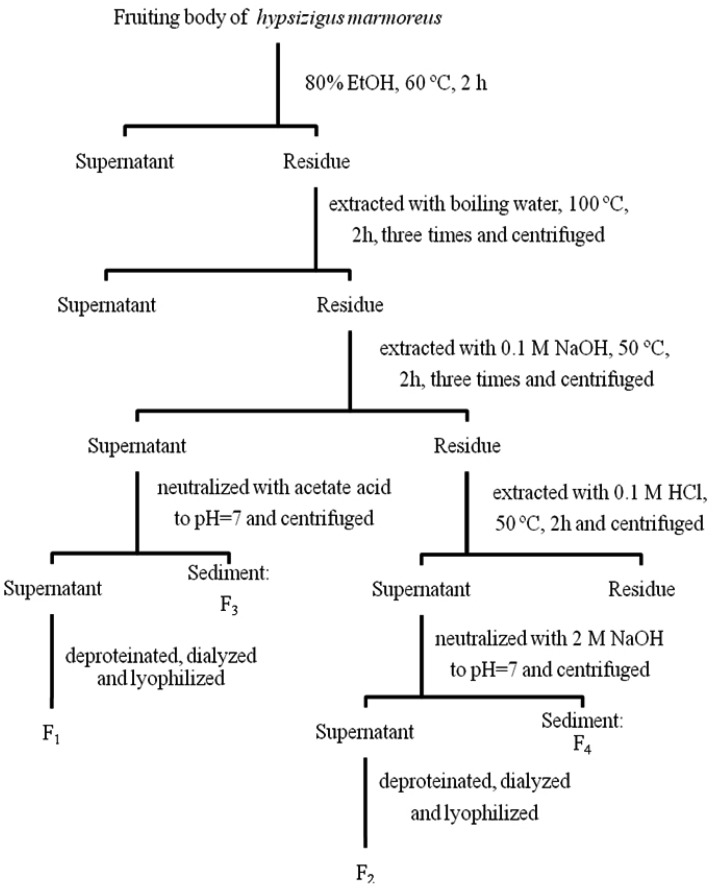
The extraction scheme used to isolate water-unextractable proteoglycans from *Hypsizygus marmoreus*.

Figure 6 shows the extraction procedure of the water-unextractable proteoglycans from *H. marmoreus*. The milled sample was refluxed with 80% ethanol (EtOH) at 60 °C for 2 h to remove the lipophilic substances and was then heated in boiling water for 2 h (×3). 

After centrifugation at 1,500 g for 10 min, the supernatant was discarded to remove the water soluble polysaccharides, and the remaining residue was dried in a vacuum oven at 25 °C. The dried biomass (10 g) was extracted with 0.1 M NaOH (500 mL) at 50 °C for 2 h (×2), followed by the centrifugation at 1,500 g for 10 min. The supernatants were collected and neutralized by acetic acid. After incubating the supernatants at 4 °C over night, the sediment was separated by centrifugation (3,000 g for 20 min) to obtain alkali-insoluble proteoglycan (F3). EtOH was added to the supernatants to get a final EtOH concentration of 70% (v/v), and then the solution was placed at 4 °C overnight. The precipitate was obtained by centrifugation at 1,500 g for 10 min and dried in a vacuum oven at 25 °C. The dried residue was redissolved in distilled water, deproteinated by the Sevag method [33], exhaustively dialyzed against distilled water and then freeze-dried to yield the alkali-soluble proteoglycan (F1). The alkali extracted residue was further extracted with 0.1 M HCl at 50 °C for 2 h, followed by centrifugation, neutralization and incubation at 4 °C overnight. This sediment produced the acid-insoluble portion of the proteoglycan (F4). The proteoglycans in the supernatant were precipitated using EtOH (70%), deproteinated by the Sevag method, dialyzed and lyophilized to yield the acid soluble proteoglycan (F2) fraction.

### 3.3. Determination of the Total Carbohydrate, Protein and Monosaccharide Composition of the Proteoglycans

The total carbohydrate and protein content of the proteoglycans were determined by the phenol-sulfuric acid method using glucose as a standard [34], and by the Lowry method [35] using a commercial assay kit (Bio-Rad, Hercules, CA, USA), respectively. The monosaccharide composition was quantitatively determined after total hydrolysis of proteoglycans (60 mg) using 2 M trifluoroacetic acid (TFA) at 120 °C for 5 h. Hydrolysate was neutralized by 2 M NaOH (final pH 5–8), evaporated with dried nitrogen stream and dissolved in 100 μL of distilled water. Sample injected into the HPLC system (eluting with 85% acetonitrile) consisting of a pump (Waters 510, Milford, MA, USA), an injection valve (Rheodyne 7010, Rohnert Park, CA, USA) with a 20-μL sample loop, a column (carbohydrate analysis column, 4.6 × 250 mm, Waters) and a refractive index (RI) detector (Waters 2414).

### 3.4. Glycosidic Linkage Analysis

The glycosidic linkage analysis of the polysaccharide moiety of the F1 and F2 fractions was carried out using the method of Hakomori [29] with slight modifications. The proteoglycans (2–3 mg) were dissolved in DMSO (0.5 mL) under nitrogen and then methylated with CH_3_I (0.3 mL) and dried NaOH powder (20 mg). Partially methylated alditol acetates were prepared from fully methylated samples by acid hydrolysis with 4 M TFA at 100 °C for 6 h followed by the reduction of the hydrolysates in water using NaBD_4_ and acetylation with acetic anhydride. The partially-methylated alditol acetates were analyzed by gas chromatography-mass spectrometry (GC-MS) (6890N/MSD 5973, Agilent Technologies, Santa Clara, CA, USA) using a HP-5MS capillary column (30 m × 0.25 mm × 0.25 μm) (Agilent Technologies) under previously reported conditions [36]. 

### 3.5. Nuclear Magnetic Resonance (NMR) Spectroscopy

For structural assignments, the spectra on the F1 and F2 fractions were recorded on a solution of 10 mg in 0.5 mL D_2_O at 50 °C. ^1^H-, ^13^C- and DEPT-135-^13^C-NMR spectra were recorded on a JEOL ECA-600 spectrometer (JEOL, Akishima, Japan), equipped with a 5 mm multi-nuclear auto-turning TH tunable probe, at a base frequency of 150 MHz for ^13^C and 600 MHz for ^1^H, respectively. Two-dimensional ^1^H-^1^H COSY, TOCSY, and ^1^H-^13^C HMQC, HMBC experiments were conducted using the pulse programs supplied with the manual.

### 3.6. Determination of the Weight Average Molecular Weight

The weight average molecular weight (M_w_) and radius of gyration (R_g_) of the proteoglycans were determined using our previously reported method [15]. Briefly, the F1 and F2 proteoglycans were dissolved in distilled water, and the F3 and F4 proteoglycans were dissolved in 0.01 N NaOH (1 mL), heated at 50 °C for 15 min, diluted with water (1 mL) and neutralized with 0.01 M HCl. The proteoglycan solution was heated in a microwave oven (RE-552W, SamSung, Seoul, Korea) for 30 s prior to analysis using high-performance size-exclusion chromatography (HPSEC) coupled with an UV (#2484, Waters, Milford, MA, USA), multi-angle laser light-scattering (MALLS) (Heleos, Wyatt Technology Corp., Santa Barbara, CA, USA) and refractive index (RI) (#2414, Waters) detection system (HPSEC-UV-MALLS-RI system) using a SEC column (TSK G5000 PW, 7.5 × 600 mm, TosoBiosep, Montgomeryville, PA, USA). The M_w_ and R_g_ values were calculated from the data collected from the MALLS and RI detectors using ASTRA 5.3 software (Wyatt Technology Corp.).

### 3.7. Assays for Macrophage Proliferation

The impact of the proteoglycans on macrophage proliferation was determined using Raw264.7 cells (ATCC, Rockville, MD, USA), following our previously described method [15]. Briefly, the cells in RPMI-1640 medium containing 10% Fetal Bovine Serum (FBS) were incubated with the sample solution at 1.5, 3, 6, or 12 μg/mL in a 96-well microplate. Control wells were treated with the medium only. Proliferation was quantified by the WST-1 assay described above.

### 3.8. Nitric Oxide Production

The effect of proteoglycans on nitric oxide (NO) release was determined by measuring the NO levels in the culture supernatant using the Griess reaction as described by Green *et al*. [37]. Raw264.7 cells (1 × 10^6^ cells/mL) were incubated in an RPMI-1640 medium containing 10% FBS for 24 h at 37 °C in the presence of 5% CO_2_. The sample solution at 1.5, 3, 6, or 12 μg/mL, or lipopolysaccharide (LPS, 1 μg/mL), which was used as a positive control, were added to the cultured cells in triplicate, and the cells were incubated for 24 h at 37 °C. After treating with Griess reagent (1% [w/v] sulfanilamide and 0.1% [w/v] *N*-[1-naphthyl] ethylenediamine hydrochloride in 2.5% [v/v] phosphoric acid), the absorbance of the solution was measured at 540 nm with an EL-800 microplate reader (BioTek instruments). NO production from the Raw264.7 cells was calculated with reference to a standard curve that was obtained with NaNO_2_ (1–200 μM in the culture medium).

### 3.9. Assay for Cytokine Secretion

The concentration of prostaglandin E_2_ (PGE_2_) and interleukin-1β (IL-1β) released from the Raw264.7 cell line after treatment with the proteoglycans was determined using an ELISA assay (Prostaglandin E_2_, EIA Kit, Assay Designs, Ann Arbor, MI, USA; Quantikine Immunoassay Mouse IL-1β, R&D Systems, Minneapolis, USA). Raw264.7 cells (1 mL, 1 × 10^6^ cells/mL) were incubated in RPMI-1640 medium containing 10% FBS with the sample solution at 1.5, 3, 6 or 12 μg/mL in a 24-well microplate. LPS (1 μg/mL) was used as a positive control. After incubation for 72 h, the amount of PGE_2_ and IL-1β in the supernatants was detected and quantified according to the manufacturer’s protocol.

### 3.10. Reverse Transcription-Polymerase Chain Reaction (RT-PCR)

Raw264.7 cells (1 × 10^6^ cells/well) were incubated at 37 °C (5% CO_2_) with LPS (1 μg/mL) and 12 μg/mL of the proteoglycans for 18 h. After incubation, total RNA was extracted from the cells using TRIzol Reagent (Invitrogen, Carlsbad, CA, USA), according to the manufacturer's protocol, and stored at −80 °C until use. The extracted RNA (2 μg) was quantified spectrophotometrically and used as templates for first strand cDNA synthesis with an oligo-(dT)_20_ primer and Superscript III Reverse Transcriptase (Invitrogen). The resulting cDNA was amplified Polymerase Chain Reaction (PCR) using GoTaq Flexi DNA Polymerase (Promega, Madison, WI, USA). Cytokine sequence amplification was conducted using several primer sequence pairs: 5′-CCCTTCCGAAGTTTCTGGCAGCAGC-3′ (forward) and 5′-GGCTGTCAGAGCCTCGTGGCTTTGG-3′ (reverse) for iNOS (GenBank accession no. NM_010927.1), 5′-CCCCCACAGTCAAAGACACT-3′ (forward) and 5′-GAGTCCATGTTCC AGGAGGA-3′ (reverse) for COX-2 (GenBank accession no. NM_011198.3), 5′-ATGAGCACAG AAAGCATGATC-3′ (forward) and 5′-TACAGGCTTGTCACTCGAATT-3′ (reverse) for TNF-α (GenBank accession no. NM_013693.1), 5′-ATGGCAACTGTTCCTGAACTCAACT-3′ (forward) and 5′-CAGGACAGGTATAGATTCTTTCCTTT-3′ (reverse) for IL-1β (GenBank accession no. NM_008361.3), 5′-TTCCTCTCTGCAAGAGACT-3′ (forward) and 5′-TGTATCTCTCTGAAGGACT-3′ (reverse) for IL-6 (GenBank accession no. NM_031168.1), and 5′-TGGAATCCTGTGGC ATCCATGAAAC-3′ (forward) and 5′-TAAAACGCAGCTCAGTAACAGTCCG-3′ (reverse) for β-actin (GenBank accession no. NM_007393.3). The PCR reactions were performed as follows: initial denaturation at 94 °C for 3 min; 30 cycles of denaturation (94 °C for 30 s), annealing (56 °C for 40 s) and extension (72 °C for 1 min); and the final extension step at 72 °C for 10 min. PCR products were electrophoresed on 1% agarose gels and visualized by ethidium bromide staining. Gels were scanned and analyzed with image analysis software (Kodak Digital Science, Kennesaw, GA, USA), and the results were expressed with relative intensity compared to β-actin. 

### 3.11. Statistical Analyses

All experiments were run in triplicate and the data are presented as the mean value with standard deviation. Statistical differences were tested using Student’s *t* test, a one-way analysis of variance (ANOVA), and Duncan's multiple-range test using Statistical Analysis System (SAS Institute, Cary, NC, USA).

## 4. Conclusions

Alkali and acid extraction procedures led to the production of four water-unextractable proteoglycans (F1, F2, F3 and F4) from *H. marmoreus*, having various chemical compositions and M_w_ values, as well as different molecular conformations. F1 and F2 fractions exhibited significant potency to induce NO and cytokine release from Raw264.7 cells, suggesting strong immunomodulating activities. The search for bioactive compounds that can boost the immune function is an important research area of immunopharmacological studies. The current study, therefore, demonstrates the potential for water-unextractable proteoglycans as medicinal, pharmacological and functional food ingredients. 
